# Supplementary material for: Facile Fabrication of Multi-Hydrogen Bond Self-Assembly Poly(MAAc-co-MAAm) Hydrogel Modified PVDF Ultrafiltration Membrane to Enhance Anti-Fouling Property
Source: Membranes (Basel). 2021 Sep 30;11(10):761. doi: 10.3390/membranes11100761 (PMC8537210; doi:10.3390/membranes11100761)
Supplement: Supplementary file 1 [file membranes-11-00761-s001.zip › membranes-1381046-supplementary-done.pdf]

membranes

Supplementary Information（SI）

Facile fabrication of multi-hydrogen bond self-assembly poly(MAAc-co-MAAm) hydrogel modified PVDF ultrafiltration membrane to enhance anti-fouling property

Weigui Fu*[1], Guoxia Li[1], Gaowei Zhai[1], Yunji Xie[2,3], Meixiu Sun[4], Patrick Théato[2,3], Yiping Zhao[1], and Li Chen[1]

[1] State Key Laboratory of Separation Membranes and Membrane Processes, School of Materials Science and Engineering, Tiangong University, Tianjin 300387, PR China

[2] Institute for Chemical Technology and Polymer Chemistry (ITCP), Karlsruhe Institute of Technology (KIT), Engesserstraße 18, 76131 Karlsruhe, Germany

[3] Soft Matter Synthesis Laboratory, Institute for Biological Interfaces 3 (IBG-3), Karlsruhe Institute of Technology (KIT), Herrmann-von-Helmholtz-Platz 1, 76344 Eggenstein-Leopoldshafen, Germany

[4] Institue of biomedical Engineering, Chinese Academy of Medical Science & Peking Union Medical College, Tianjin 300192, PR China

* Address corresponding to E-mail: tjfwg@hotmail.com

1. Optimum grafting conditions

Firstly, the modified membranes were prepared the same molar ratio, different monomer concentration to obtain optimal grafting degrees (Table S1). Water contact angle (WCA) was used to characterize the hydrophilicity of the membranes. As shown in Figure S1, when the grafting degree of membranes increased from 0 mg/cm^2 (M0) to 2.4 mg/cm^2 (M5), the corresponding WCA decreased obviously from 120.6° ± 2.1° to 35.3° ± 1.8°. Although M4 and M5 had different grafting degrees, their contact angles were similar, that is to say, the hydrophilicity of the two membranes was similar. Considering the holes of the membrane surface would be blocked under high grafting degree, the grafting degree of M4, about 2.1 mg/cm^2, was selected as the optimal grafting degree for all the modified membranes with different monomer molar ratios.

Table S1 Weight ratio of monomer solutions for the PVDF modified membranes.

Membranes	MAAc (g/L)	MAAm (g/L)	$(NH_4)_2Fe(SO_4)_2{\cdot}6H_2O$ (g/L)	Grafting degree (mg/cm^2)
M0	--	--	--	--
M2	4.0	4.0	2.0	1.1
M3	6.0	6.0	3.0	1.6
M4	8.0	8.0	4.0	2.1
M5	10.0	10.0	5.0	2.4

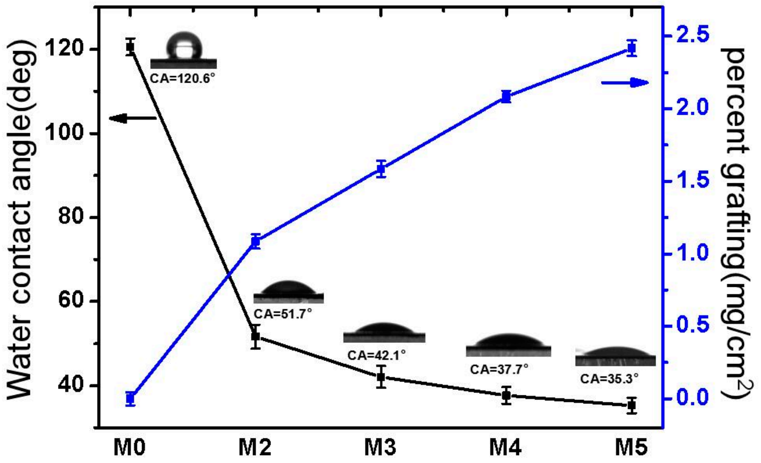

Figure S1. Water contact angle and grafting degree of different membranes.

2. Roughness of membrane surfaces

AMF images of the membrane surfaces grafted a monomer MAAc or MAAm, separately, with the same grafting degree (0.6 mg/cm^2) were shown in Figure S2. It shows that the roughness of PVDF-g-MAAm (175±11 nm) is much higher than that of PVDF-g-MAAc (146±15 nm). It may be because the reaction activity of COOH groups is higher, thus forming higher grafting density of MAAc on the membrane surface.

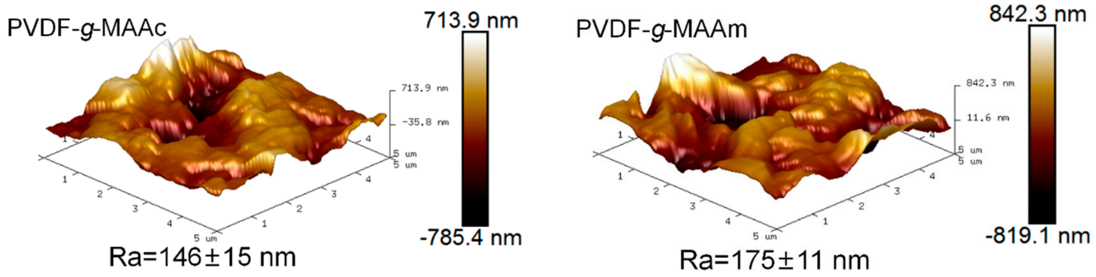

Figure S2. AFM photographs of the modified membranes with a monomer.

3. Porosity and mean pore size of membranes

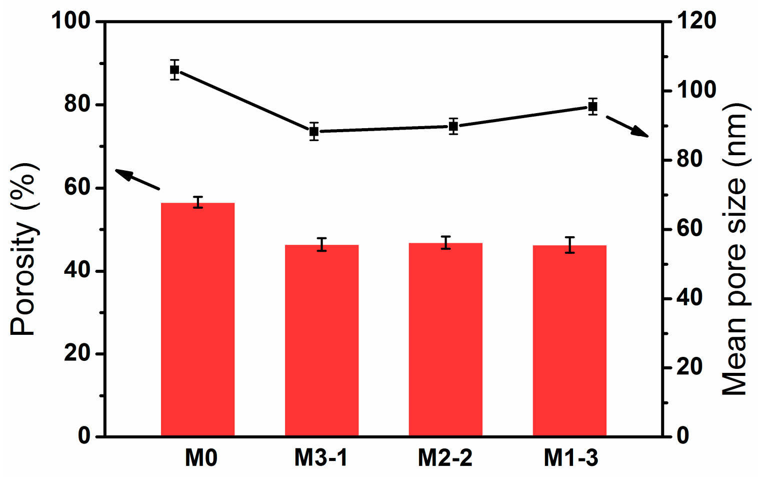

Figure S3. Porosity and mean pore size of the membranes.

4. Particle size distribution of foulants

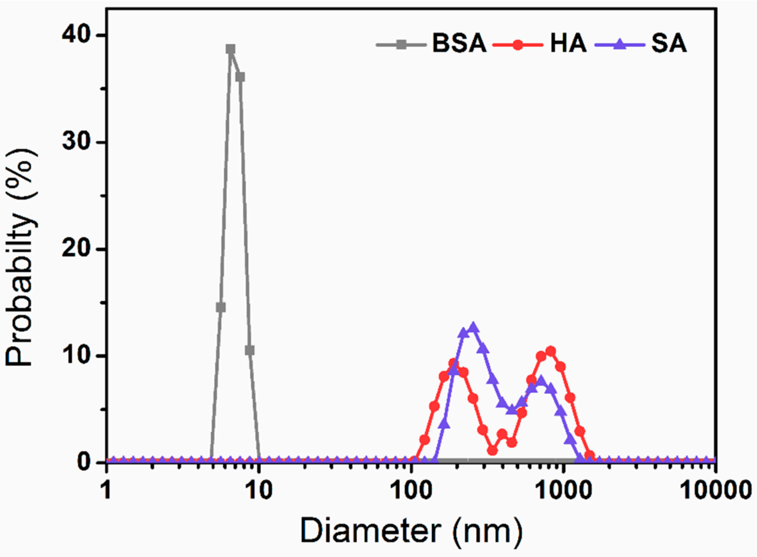

Figure S4. Particle size distribution of foulants.

5. Stability of gel layer

Table S2 Membrane weight before and after ultrasonic and immersion treatment (pH=2, 9) for 30 min, respectively.

M2-2	Ultrasonic 30 min	Immersion 30 min (pH=2)	Immersion 30 min (pH=9)
Pre-test status (mg)	144.3 ± 0.1	125.5 ± 0.2	125.3 ± 0.2
Post-test status (mg)	144.0 ± 0.2	125.1 ± 0.4	125.1 ± 0.1

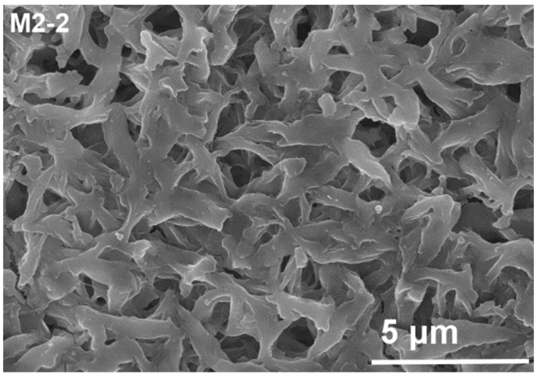

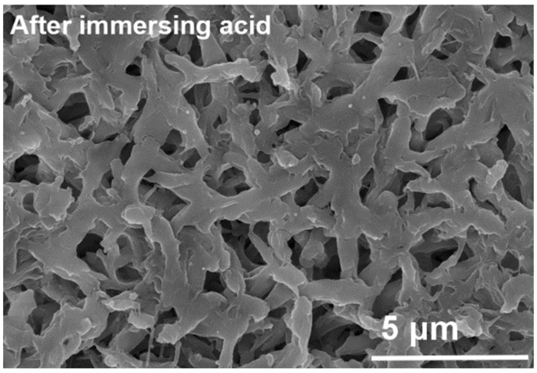

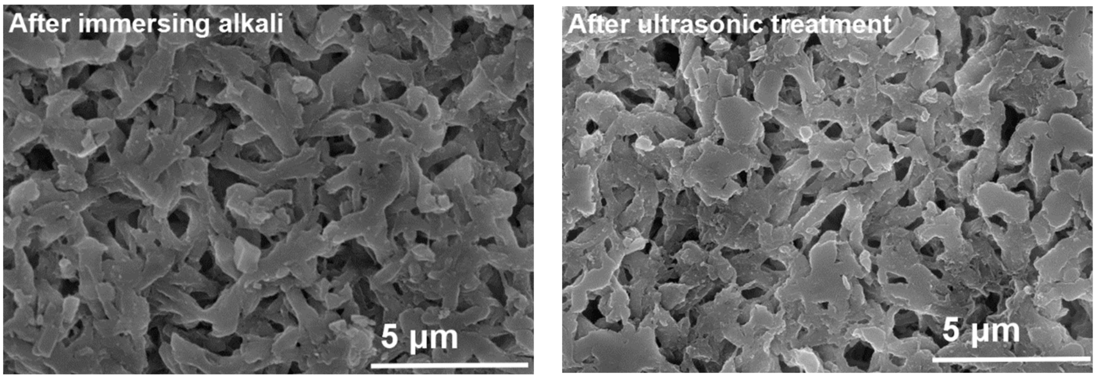

Figure S5. SEM images (× 7,000) of the M2-2 modified membrane before and after ultrasonic treatment, soaking in acid (pH=2) or alkaline (pH=9) aqueous solution.

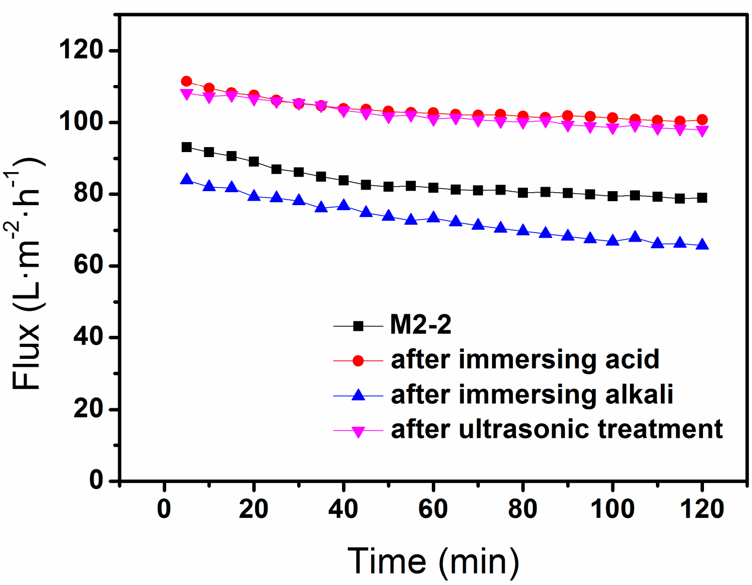

Figure S6. The flux of the membranes before and after ultrasonic treatment or soaking (pH=2, 9).
